# Auditory presentation and synchronization in Adobe Flash and HTML5/JavaScript Web experiments

**DOI:** 10.3758/s13428-016-0758-5

**Published:** 2016-07-15

**Authors:** Stian Reimers, Neil Stewart

**Affiliations:** 1 Department of Psychology, City University London, Northampton Square, London EC1V 0HB UK; 2 Department of Psychology, University of Warwick, Coventry, UK

**Keywords:** Web, Internet, Audio, Synchronization, JavaScript

## Abstract

Substantial recent research has examined the accuracy of presentation durations and response time measurements for visually presented stimuli in Web-based experiments, with a general conclusion that accuracy is acceptable for most kinds of experiments. However, many areas of behavioral research use auditory stimuli instead of, or in addition to, visual stimuli. Much less is known about auditory accuracy using standard Web-based testing procedures. We used a millisecond-accurate Black Box Toolkit to measure the actual durations of auditory stimuli and the synchronization of auditory and visual presentation onsets. We examined the distribution of timings for 100 presentations of auditory and visual stimuli across two computers with difference specs, three commonly used browsers, and code written in either Adobe Flash or JavaScript. We also examined different coding options for attempting to synchronize the auditory and visual onsets. Overall, we found that auditory durations were very consistent, but that the lags between visual and auditory onsets varied substantially across browsers and computer systems.

**Electronic supplementary material:**

The online version of this article (doi:10.3758/s13428-016-0758-5) contains supplementary material, which is available to authorized users.

The goal of many experiments in the behavioral sciences is to present stimuli to participants for a known, accurate amount of time, and record response times (RTs) to those stimuli accurately. Sometimes, multiple stimuli have to be synchronized or presented with known, accurate offsets, and multiple responses, such as sequences of keypresses need to be recorded. As much research is now conducted online, many researchers have examined the extent to which experiments requiring accurate presentation durations or RTs are feasible using standard Web-based technologies such as Adobe Flash and JavaScript (for an overview of the various ways of running Web-based RT experiments, see Reimers & Stewart, 2015).

Two broad approaches have generally been used. The first is to attempt to compare results from human participants completing an experiment online and in a more traditional lab-based setting, either by attempting to replicate well-established lab-based effects online, or by running lab- and Web-based versions of the same study. Here, the results from lab- and Web-based versions of a given study have been largely comparable (e.g., Crump, McDonnell, & Gureckis, 2013; de Leeuw & Motz, 2016; Reimers & Stewart, 2007; Schubert, Murteira, Collins, & Lopes, 2013; Simcox & Fiez, 2014), although under some boundary conditions with short presentation durations and complex learning tasks, Web-based performance has been inconsistent with the lab results (Crump et al., 2013). For a discussion of this approach and some of its advantages and disadvantages, see Plant (2016) and van Steenbergen and Bocanegra (2015).

The second broad approach has been to compare directly the accuracy of browser-based stimulus presentation and RT recording using specialist software or hardware (e.g., Neath, Earle, Hallett, & Surprenant, 2011; Reimers & Stewart, 2015; Schubert et al., 2013; Simcox & Fiez, 2014). In general, visual stimulus presentation durations are longer than specified in the code to control their presentation, and show some quantizing, presumably linked to a monitor’s refresh rate.

## Auditory stimuli through a Web browser

Almost all existing Web-based RT research has used individual visually presented stimuli. There are several likely reasons for this. First, it reflects a focus in cognitive psychology directed more toward visual rather than auditory perception and processing. (To illustrate, in the UK, the core introductory cognitive psychology textbooks have several chapters on aspects of visual processing, but only a few pages on auditory perception.)

Second, there may have been more practical barriers to running Web-based experiments with auditory stimuli. The ability for users to see visually presented stimuli is a given, as all computers use a visual interface—that is, a monitor—for interaction. Audio has traditionally been more optional: Early PCs could only produce simple beeps via the internal speaker, and only a little over a decade ago, many business PCs included sound cards only as an optional extra. A more recent issue has been user-driven: people do not always have the ability to listen to sounds presented on their computer, lacking speakers or headphones. However, the increasing use of the Web to play video, and applications requiring audio such as skype, is likely to have made the ability to use audio more widespread.

Third, researchers have legitimate concerns about the uncontrolled environment in which Web-based studies are run (for a more detailed overview, see Slote & Strand, 2016). Although the appearance of visual stimuli varies substantially from system to system, in terms of size, hue, saturation, contrast, among other things, the fact that people need to be able to view a display in order to use a computer means that the basic properties of a stimulus will be perceived by a participant. On the other hand, auditory stimuli may be too quiet to be perceived; they may be distorted; they may be played in a noisy environment making their discriminability impossible. They may also be presented monaurally or binaurally, which can affect perception (Feuerstein, 1992), in mono or stereo (which would affect dichotic listening tasks), and so on.

Fourth, the presentation of browser-based audio is generally more complicated than the presentation of visual stimuli. For example, no single audio format is supported by all current popular PC browsers, and until the recent development of the HTML5 standards, the optimal methods for playing audio varied by browser (for an introduction to earlier methods for presenting audio, see Huckvale, 2011).

Finally, there are concerns regarding the variability in audio presentation onset times. Most notably, Babjack et al. (2015) reported substantial variability in the latency between executing the code to present a sound, and that sound being presented. In their research, a Black Box ToolKit (see below) was used to measure the latency between pulse generated by the testing system, which would be detected immediately, and a sound that was coded to begin at the same time. The results showed that the mean latency varied substantially across different hardware and software combinations, from 25 to 126 ms of milliseconds, and that one-off latencies could be as much as 356 ms.

### Existing research

Experiments using auditory stimuli in Web-based studies started in the very earliest days of internet-mediated research (Welch & Krantz, 1996). However in the intervening 20 years, very little published research appears to have presented auditory stimuli over the Web (for overviews, see Knoll, Uther, & Costall, 2011, and Slote & Strand, 2016), and less still has examined the accuracy of doing so, systematically.

Some existing research has used long audio files embedded in a webpage (e.g., Honing, 2006) or downloaded to the user’s computer (e.g., Welch & Krantz, 1996). Auditory stimuli have included excerpts of musical performance (Honing, 2006), unpleasant sounds such as vomiting and dentists’ drills (Cox, 2008), and speech (Knoll et al., 2011; Slote & Strand, 2016).

In Knoll et al.’s (2011) study, participants listened to 30-second samples of low-pass filtered speech, spoken by a UK-based mother (a) to her infant, (b) to a British confederate, and (c) to a foreign confederate. Participants made a series of affective ratings for each speech clip. The experiment was run in two conditions: One was a traditional lab-based setup; the other used participants recruited and tested over the Web. The pattern of results was very similar across the two conditions, with participants in both conditions rating infant-directed speech as more positive, more comforting and more encouraging than either of the two other speech types.

More recently, Slote and Strand (2016) examined whether it would be possible to measure RTs to auditory stimuli online. In their Experiment 1, participants were presented with auditory consonant-vowel-consonant words such as “fit.” In the identification condition, participants had to identify and type in the word presented with a background babble added. In the lexical decision condition, participants made speeded word–nonword judgments to the same words and matched nonwords such as “dak.” The experiment was run both in the lab and over the Web using JavaScript, with participants recruited through Amazon Mechanical Turk. In the identification task, performance was significantly better in the lab condition than the Web condition; however, the correlation between item-level identification accuracy in the two conditions was very high (*r* = .89). (Similar correlations between lab- and Web-based auditory identification performance with have been reported by Cooke, Barker, Garcia Lecumberri, & Wasilewski, 2011.) Most interestingly, the correlation between lexical decision times across the two conditions was also very high (*r* = .86). This was numerically higher than the split-half correlation within the lab condition, suggesting that a Web-based methodology was as capable as a lab-based methodology for discriminating between stimuli of differing difficulties, as captured in RTs.

To examine the accuracy of RTs to auditory stimuli directly, Slote and Strand (2016) ran a second experiment, this time using specialist hardware to generate accurate, known response times to auditory stimuli. They used two different JavaScript methods, the Date method and the Web Audio application program interface (API; see below), to present auditory sinusoidal stimuli and record RTs, which they compared against the actual RTs measured by the specialist hardware attached to another computer. They found that the recorded RTs were between 54 ms (Web Audio) and 60 ms (Date method) longer than the actual RTs, presumably reflecting a combination of lag to presentation and the time taken for a keypress to be detected and acted upon by JavaScript. Crucially, they also reported that the standard deviation for these overestimates was generally small—between 4 and 6 ms. Finally, they found that the Date method was susceptible to processor load, with variability increasing when substantial extra concurrent processing load was applied to the system.

## Research rationale

The aim of the studies reported here was to extend the existing work on the use of auditory stimuli in Web-based research. One aim was to examine variability in the duration of auditory stimuli presented through a browser. Given the intrinsic temporal nature of auditory stimuli, we would expect durations to be consistent, but we tested this directly. The main aim was to examine whether it is possible to synchronize auditory and visual presentation using JavaScript or Flash. Researchers on many areas have examined cross-modal perception: the influence of stimuli presented in one modality on perception in another modality. Most famous is the McGurk effect (McGurk & MacDonald, 1976), in which watching a person articulate /ga/ at the same time as hearing /ba/ leads to the perception of /da/.

Although some of the best-known effects tend to be based on complex dynamic visual stimuli like mouthed speech, presented as video clips, others are based on simpler stimuli. For example, the ventriloquist effect, in which the perception of the location of sounds is affected by the location of concurrent visual stimuli, is examined using simple stimuli such blobs of light and clicks of sound presented concurrently or asynchronously (e.g., Alais & Burr, 2004). Similarly, emotion judgments to static monochrome images of faces are affected by the tone of voice in which irrelevant auditory speech is presented (de Gelder & Vroomen 2000). Synchronized bimodal presentation of auditory and visual words is also used to examine language comprehension processes (e.g., Swinney, 1979), and abstract stimuli such as tones and visual symbols, varying in synchronization, have been used in research on attention (Miller, 1986). For many, though not all, of these tasks, tight control must be kept on the synchronization of auditory and visual stimulus onset times. For example, the McGurk effect is reduced substantially if the auditory onset occurs more than 30 ms before the visual onset (van Wassenhove, Grant & Poeppel, 2007).

For this research, we were primarily interested in the extent to which control of auditory and visual stimulus onset asynchronies (SOAs) could be maintained over the Web across different system–browser combinations. We were less interested in the absolute SOA, because a consistent SOA can be corrected by adding a delay to the presentation of the stimuli on one of the modalities. However, substantial variability in SOAs across computer hardware and software combinations would be a much more difficult problem to solve.

The second aim was to examine indirectly how accurate measured RTs to auditory stimuli might be. We had previously shown the degree to which JavaScript and Flash overestimate visual RTs, in part as a result of a lag between the instruction for a stimulus to be presented and the stimulus’s appearance on the computer monitor. If we attempted to present an auditory and visual stimulus concurrently, we could use the measured SOAs, combined with the known overestimation of RTs to visual stimuli that had previously been reported, to calculate the expected overestimation of RTs to auditory stimuli.

In the studies reported here, we used the two programming approaches generally used for running RT experiments online: JavaScript and Adobe Flash. JavaScript, coupled with HTML5 and CSS3, is emerging as the standard for running Web-based RT studies in the behavioral sciences (e.g., Crump et al., 2013; de Leeuw & Motz, 2016; Hilbig, 2015), and several libraries have recently been developed to help researchers set up experiments using JavaScript (e.g., jsPsych: de Leeuw, 2015; psiTurk: Gureckis et al., 2015; and QRTEngine: Barnhoorn, Haasnoot, Bocanegra, & van Steenbergen, 2015). Although Flash is, for many understandable reasons, waning in popularity, it has been, and is still, used in behavioral research (e.g., Project Implicit, n.d.; Reimers & Maylor, 2005; Reimers & Stewart, 2007, 2008, 2015; Schubert et al., 2013). Other programming languages, such as Java, are now only rarely seen in online behavioral research, in part because of security concerns (though see Cooke et al., 2011, for an example using auditory stimuli). Both Flash and JavaScript are capable of presenting auditory stimuli.

The basic designs of all studies were identical: We aimed to present a visual stimulus (a white square) and an auditory stimulus (a 1000-Hz sine wave^1^) on the screen for 1,000 ms, with concurrent onsets of the two stimuli. We would repeat this 100 times, and then would report, across a series of browser and computer system combinations, the distribution of SOAs between the visual and auditory onsets, along with the visual and auditory durations, to see how they deviated from the desired performance, and, crucially, how much they varied across different system–browser combinations.

The implementation was designed along lines similar to those of Reimers and Stewart (2015). We used the Black Box ToolKit, Version 2 (www.blackboxtoolkit.com; see also Plant, Hammond & Turner, 2004), to measure accurately the onsets and durations of visual and auditory stimuli. To do this, we attached one of the toolkit’s opto-detectors to the monitor at the location where the white square would appear, and placed the toolkit’s microphone next to a set of USB headphones or by the computer’s speaker. The toolkit recorded the onsets and offsets of auditory and visual stimuli, and detection thresholds were set at the start of each session.

## Study 1

There are several ways of coding and synchronizing auditory and visual stimulus generation. In Study 1, we used the simplest, unconstrained approach, in which the computer code essentially simultaneously executed commands to present visual and auditory stimuli. The basic approach is shown in this pseudocode:

1. Begin a new trial with a black screen
2. Present the white square on the screen
3. Start a 1,000-ms timer
4. Play the 1,000-ms sine wave
5. When 1,000-ms timer completes, hide rectangle
6. Wait 500 ms, and repeat

Thus, here we simply sent practically concurrent requests for audio and visual stimuli to start. The code was implemented in Flash (using ActionScript 3, passing an embedded mp3 sample to a SoundChannel) and JavaScript (using the HTML5 <audio> tag, and the JavaScript play() method). We used two systems: a reasonably well-powered desktop PC (Dell OptiPlex 9010 running Win 7, i3-3220, 8Gb, Intel HD 2500, with Dell P2211H monitor), and an often frustratingly underpowered touchscreen laptop (Lenovo 14-in. Flex 2 running Win 10, Pentium 3558U, 4 GB, Intel HD Haswell). On each system, we tested the three major browsers: Google Chrome, Mozilla Firefox, and Microsoft Internet Explorer (Microsoft Edge for the Win 10 laptop). We used an mp3 file for the audio, because this was the only format compatible with all browsers in our test.

### Results

The means, *SD*s, and ranges for each of the conditions are given in Table 1.

**Table 1:** Deviations from intended stimulus durations and synchronization by browser and system in Study 1, for desktop PC (top) and laptop (bottom)

	JavaScript			Flash		
	Microsoft	Firefox	Chrome	Microsoft	Firefox	Chrome
Desktop						
Auditory Duration						
Mean (*SD*)	+24.0 (4.1)	+19.7 (0.13)	+19.7 (0.10)	+19.7 (0.09)	+19.7 (0.15)	+19.7 (0.09)
Range	+20, +33	+20, +20	+20, +20	+20, +20	+20, +21	+20,+20
Visual Duration						
Mean (*SD*)	+36.1 (3.6)	+21.4 (4.3)	+23.9 (6.9)	+18.9 (5.0)	+21.4 (9.6)	+17.4 (1.6)
Range	+20, +37	+20, +37	+20, +37	+17, +34	+1, +51	+17, +34
SOA (Auditory – Visual)						
Mean (*SD*)	+34.5 (4.3)	+60.6 (7.2)	+31.2 (5.1)	+81.8 (10.0)	+77.3 (10.2)	+104.1 (6.7)
Range	+25, +41	+45, +74	+22, +42	+22, +104	+28, +104	+103,+114
Laptop						
Auditory Duration						
Mean (*SD*)	+21.5 (0.61)	+13.2 (5.0)	+21.8 (0.77)	+21.7 (0.72)	+22.0 (0.70)	+21.9 (0.69)
Range	+21, +23	+0, +16	+21, +24	+21, +23	+21, +23	+21, +23
Visual Duration						
Mean (*SD*)	+60.2 (4.5)	+48.0 (7.2)	+62.3 (15.1)	–11.1 (4.4)	+32.1 (0.70)	–4.5 (10.9)
Range	+52, +78	+36, +77	+33, +110	–13, +13	+3, +52	–30, +29
SOA (Auditory – Visual)						
Mean (*SD*)	+34.5 (2.1)	+60.7 (13.1)	+55.9 (10.9)	+59.5 (8.6)	+98.7 (11.6)	+95.4 (15.8)
Range	+24, +43	+45, +181	+32, +70	+42, +79	+30, +118	+72, +210

Positive values for SOA indicate that audio lagged behind visual. The default Microsoft browser was used for both systems: internet explorer on the desktop and edge on the laptop

#### Auditory duration

The duration of auditory stimuli was very consistent, both within and across system–browser–coding configurations. Across conditions, the mean auditory duration varied by a maximum of 11 ms, and the *SD*s within a condition were generally under 1 ms. Some of the between-condition variability might also be attributable to trigger thresholds for the Toolkit’s audio detector. The overall duration was significantly longer than the 1,000-ms sound duration. However, we suspect that this may have been due to a “pop” occurring at the end of the sine wave, which increased the duration slightly. Overall, this test provided good evidence that auditory durations are consistent.

#### Visual duration

Visual presentation durations were similar to those reported by Reimers and Stewart (2015). In nearly all conditions the visual durations were longer than specified, generally by around 20 ms, although there was some variability on the laptop. For one of our tests we used the same system tested by Reimers and Stewart (2015), allowing a direct comparison to be made. Here we found that mean visual durations were longer by 19 ms (Flash) and 27 ms (JavaScript). On the same system, Reimers and Stewart (2015) reported mean durations that were longer than specified by 18 ms (Flash) and 21 ms (JavaScript).

#### SOA between auditory and visual onset

Although the code for presenting the visual and auditory stimuli was run at essentially the same time, the onsets of the visual and auditory stimuli were not concurrent. Using JavaScript, the auditory onset lagged behind the visual onset by between 35 and 61 ms. When we used Flash, the SOA was even more pronounced: between 60 and 104 ms.

#### Test–retest consistency

**Fig. 1:**
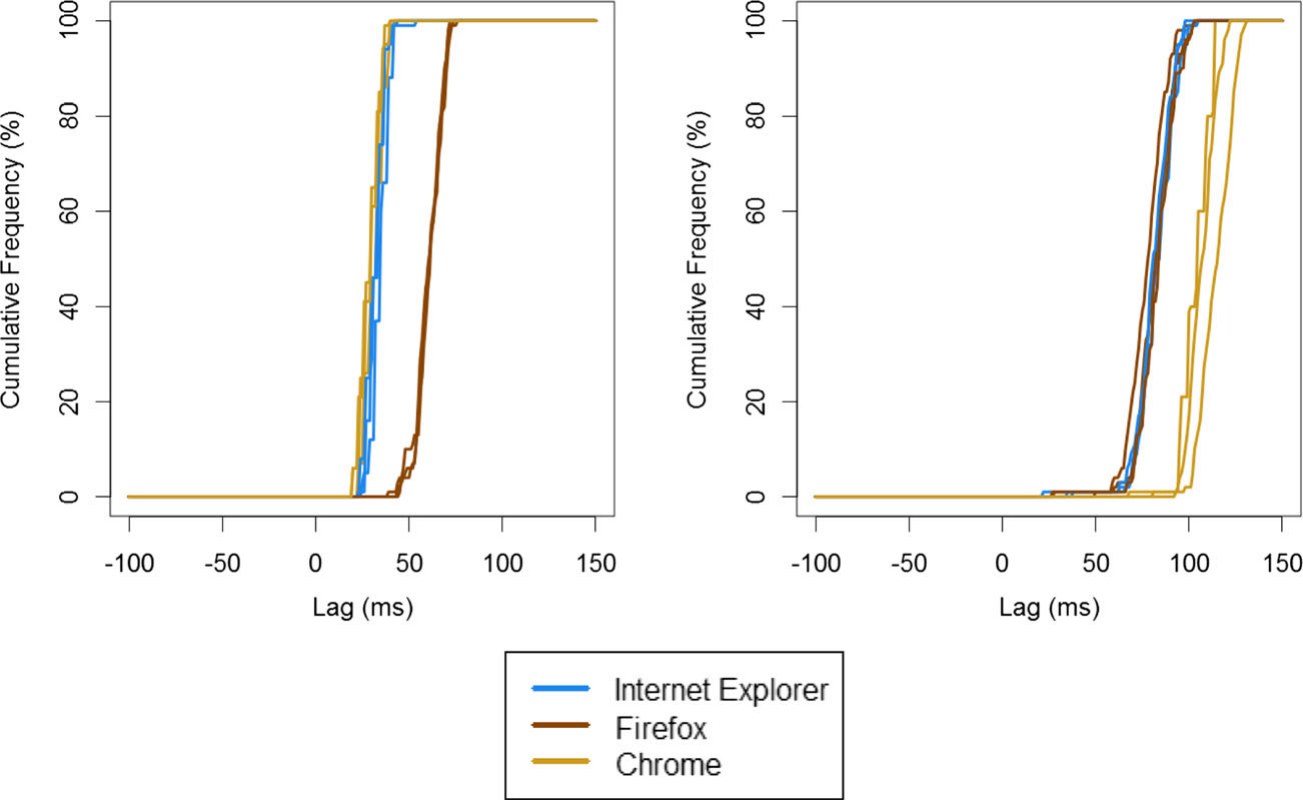
Cumulative frequency distribution of stimulus onset asynchronies (SOAs) in Study 1 across three browsers, showing test–retest consistency for three runs of 100 trials on the desktop PC. The left panel shows JavaScript performance, and the right panel shows Flash performance

It was unclear from these results whether the differences between conditions were due to something intrinsic to the system–browser combination used or could have been due to random fluctuation in performance, depending on which other processes were active at the time of running the test. In other words, were the differences across conditions reliable, or were they random noise? We therefore repeated the tests on one of the machines (the desktop PC). The results can be seen in Fig. 1. SOAs were fairly consistent across the tests of both JavaScript and Flash. This suggests that a substantial proportion of the variability across conditions was due to stable differences in performance in different browsers and systems.

### Discussion

Study 1 provided data on the accuracy of auditory durations and auditory–visual synchronization under JavaScript and Flash. Although presentation duration accuracies were very consistent, the SOA between visual and auditory stimuli was at least 35 ms. If this were a consistent SOA, it need not be a problem, because a delay could be introduced in the visual presentation to bring the two modalities into synch. However, the SOA varied with the system–browser combination used, by up to 25 ms under JavaScript, and up to 45 ms under Flash. Furthermore, this variability was not consistently attributable to a single source—say, different browsers. For example, the SOA under JavaScript and Chrome was twice as high when running on the laptop as on the desktop PC, whereas the SOAs for Firefox were very consistent across systems. As such, it would be hard to compensate for the SOA systematically.

Although the evidence from Study 1 gives some cause for concern about the synchronization of auditory and visual stimuli, there are many different ways of coding the stimulus presentation, and it could be that whereas the particular approach used here produced significant variability in SOAs, others might not. We investigated this now.

## Study 2

In this study, we attempted to reduce the variability in SOAs between auditory and visual stimuli across browsers and systems, by using a different approach. In Study 1, the code we wrote merely requested that the auditory and visual stimuli to be presented concurrently, without monitoring when the stimuli were actually presented. As we discussed above, previous research suggested that there can be a nontrivial lag between executing a command to present an audio stimulus and the stimulus’s onset, so the overall finding of a substantial lag between visual and auditory onsets was perhaps not surprising.

In Study 2, we used JavaScript or Flash start a sound playing, and then used an event listener, a procedure that runs when triggered by an event such as a mouse click or a screen refresh, to check whether the sound is actually reported as playing. As soon as it was detected as playing, the visual stimulus was presented. This gives less control over the precise point at which a stimulus starts playing, but it may reduce cross-modal asynchrony. The design is given in the following pseudocode:

1. Begin a new trial with a black screen
2. Play a sine wave mp3 lasting 1,000 ms
3. Monitor whether a sound is playing
4. If a sound is playing, make white square visible
5. Monitor whether a sound is complete
6. If the sound is complete, make white square invisible
7. Wait 500 ms, and repeat

In JavaScript, the command to make the square visible was bound to the “play” event for the sine wave. In Flash, an on-interframe Event Listener monitored the position of the playhead in a sound channel. When the playhead’s position was greater than 0—that is, when the sound was playing—the square was made visible.

### Results

**Fig. 2:**
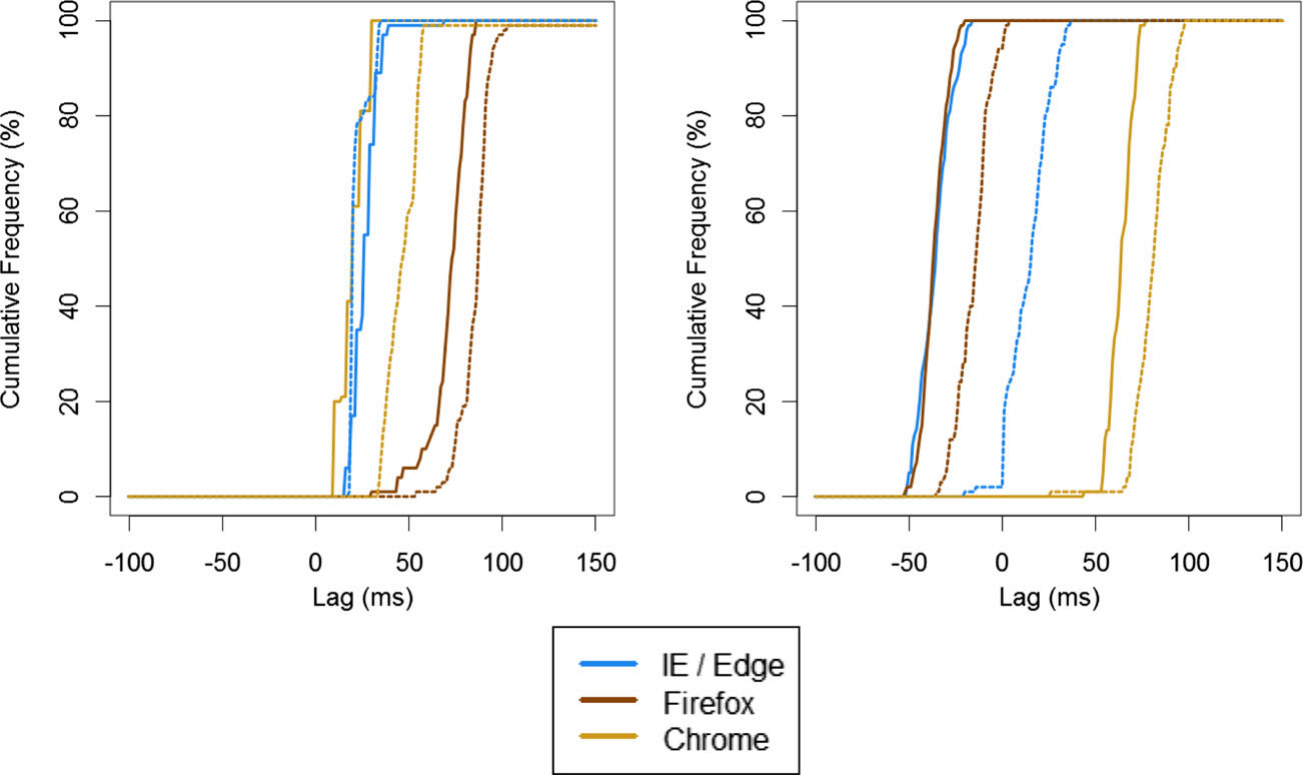
Cumulative frequency distribution of SOAs in Study 2 across three browsers, two implementations and two computer systems. The left panel shows JavaScript performance, and the right panel shows Flash performance. Solid lines show performance on the desktop PC, and the dotted lines show performance on the laptop

The results can be seen in Table 2, and the cumulative distribution of SOAs can be seen in Fig. 2.

**Table 2:** Deviations from intended stimulus durations and synchronization by browser and system in Study 2, for desktop PC (top) and laptop (bottom)

	JavaScript			Flash		
	Microsoft	Firefox	Chrome	Microsoft	Firefox	Chrome
Desktop						
Auditory Duration						
Mean (*SD*)	+25.1 (4.7)	+19.7 (0.11)	+19.7 (0.09)	+19.7 (0.09)	+19.7 (0.10)	+19.7 (0.09)
Range	+20, +34	+20, +20	+20, +20	+20, +20	+20, +20	+20,+20
Visual Duration						
Mean (*SD*)	+118.0 (8.1)	+224.3 (23.2)	+83.0 (7.2)	–13.6 (11.7)	–12.0 (11.3)	+61.7 (8.7)
Range	+103, +170	+136, +319	+69, +103	–32, +18	–33, +1	+34, +84
SOA (Auditory – Visual)						
Mean (*SD*)	+26.2 (7.2)	+71.3 (10.3)	+19.5 (6.7)	–36.1 (8.7)	–36.7 (6.7)	+63.5 (6.3)
Range	+15, +69	+30, +86	+10, +30	–52, –16	–52, –21	+43, +76
Laptop						
Auditory Duration						
Mean (*SD*)	+22.6 (4.7)	+8.1 (6.5)	+22.6 (0.20)	+22.3 (0.84)	+22.5 (0.75)	+22.4 (0.46)
Range	+20, +34	–2, +15	+22, +23	+21, +25	+21, +27	+21, +23
Visual Duration						
Mean (*SD*)	+159.2 (11.2)	+199.0 (21.7)	+120.0 (14.4)	+24.1 (14.0)	–10.4 (10.2)	+58.2 (11.9)
Range	+138, +188	+153, +271	+98, +220	–13, +54	–33, +19	+4, +84
SOA (Auditory – Visual)						
Mean (*SD*)	+21.8 (5.1)	+86.1 (12.5)	+47.1 (13.5)	+13.9 (11.2)	–15.5 (8.8)	+80.3 (9.9)
Range	+18, +34	+53, +182	+33, +158	–21, +36	–36, +3	+25, +98

Positive values for SOA indicate that audio lagged behind visual. The default Microsoft browser was used for both systems: internet explorer on the desktop and edge on the laptop

#### Auditory duration

As before, the consistency of the auditory presentation durations was very good. The results are very similar to those found in Study 1.

#### Visual duration

The binding of the visual stimulus onset and offset to the audio onset and offset led to substantial variability in visual durations. These included presentation durations that were slightly below the desired 1,000 ms with Flash and some very large excessive durations under JavaScript.

#### SOA between auditory and visual onset

The SOAs here were no better than those in Study 1, and variability across system–browser combinations was higher.

### Discussion

In this study, we used event listeners to bind the onset of the visual stimulus to the presentation of the auditory stimulus. The aim was to circumvent the well-known issue of unmeasurable lags between command execution and the actual onset of an auditory stimulus. It did not work.

This failure is perhaps not surprising: A substantial amount of sound processing is devolved to a computer’s sound card, and browser-based code has limited access to system-related information. As such, it appears that what we monitored was not the exact onset of the auditory stimulus, but either some proxy within the JavaScript or Flash environment, which may not have been related to the actual behavior of the soundcard, or, at least in some cases, a report from the soundcard that was subject to delay and variability in the timing of its presentation to the runtime environment of the JavaScript or Flash code.

It also seems clear, particularly in JavaScript, that the event triggered by sound *completion* occurred some time after the sound had finished, meaning that the visual stimulus stayed on the screen for substantially longer than it should. Overall, then, this approach appears to be no better, and may be worse, than that in Study 1.

## Study 3

In Study 3, we tried two further techniques to reduce visual–auditory SOA variability across browsers and systems. We focused on JavaScript, which is used more extensively for psychological research, and which has several different methods for controlling the presentation of audio. The first was to use a different sound format. In Studies 1 and 2, we chose to encode our sine wave as an mp3, because it is the only format that is compatible with the three major PC browsers tested here. However, mp3 is a highly compressed format, and the time taken to decompress an mp3 file before playing it may contribute to cross-browser or cross-system variability in SOAs. Furthermore, many mp3 encoders, including the one we used, add a leading 50 ms of silence to an encoded mp3 file. (The reasons for this are complex, but an overview can be seen here: http://lame.sourceforge.net/tech-FAQ.txt). Depending on the codec used to decompress the file, it appears that this initial 50 ms of silence may be stripped out, or, potentially, may remain. As such, using a different format may improve performance. On the downside, this means that it was not possible to use Microsoft Internet Explorer, because it does not currently support the playing of wav files via HTML5 and JavaScript.

The second option we tried, in our attempt to improve performance, was to use the Web Audio API for JavaScript to control the sound (see Slote & Strand, 2016, for an overview). As Slote and Strand noted, Web Audio gives access to a computer’s soundcard’s own clock, which may allow more accurate and less variable timings. Thus, in this study, we used the Web Audio API, initializing an audio context and then connecting the sine wave source to the context, before playing (using code based on that to be found in this tutorial: www.html5rocks.com/en/tutorials/webaudio/intro/), at the same time as making the square visible. Visual offset was triggered by the ending of the audio source. For the full code, see the supplementary materials. We ran two versions of this code, one for an mp3 file and one for a wav file.

### Results

The full results can be seen in Table 3.

**Table 3:** Deviations from intended stimulus durations and synchronization by browser and system in Study 3, for desktop PC (top) and laptop (bottom)

	JavaScript Web Audio mp3			JavaScript Web Audio wav		
	Edge	Firefox	Chrome	Edge	Firefox	Chrome
Desktop						
Auditory Duration						
Mean (*SD*)	-	+19.7 (0.09)	+19.7 (0.15)	-	+19.7 (0.12)	+19.7 (0.10)
Range	-	+20, +20	+20, +21	-	+20, +20	+20, +20
Visual Duration						
Mean (*SD*)	-	+137.0 (8.2)	+22.0 (8.5)	-	+66.5 (8.1)	+18.5 (9.2)
Range	-	+117, +151	+1, +34	-	+51, +85	+1, +35
SOA (Auditory – Visual)						
Mean (*SD*)	-	+45.4 (5.1)	+16.8 (5.7)	-	–5.0 (5.2)	+13.1 (4.7)
Range	-	+18, +55	+2, +25	-	–20, +5	+4, +21
Laptop						
Auditory Duration						
Mean (*SD*)	+20.1 (0.13)	+20.1 (0.12)	+20.1 (0.15)	+20.1 (0.13)	+20.1 (0.13)	+20.1 (0.13)
Range	+20, +20	+20, +20	+20, +21	+20, +20	+20, +21	+20, +20
Visual Duration						
Mean (*SD*)	+73.8 (8.3)	+114.8 (7.0)	+16.7 (7.0)	+4.7 (7.7)	+55.2 (8.1)	+16.3 (5.2)
Range	+68, +102	+101, +135	+1, +34	0, +34	+50, +85	0, +34
SOA (Auditory – Visual)						
Mean (*SD*)	+56.3 (3.9)	+32.0 (5.5)	+30.6 (5.4)	+17.6 (3.7)	–5.4 (6.3)	+33.4 (5.3)
Range	+50, +82	+23, +44	+14, +42	+13, +47	–16, +26	+15, +43

Positive values for SOA indicate that audio lagged behind visual

#### Auditory duration

The results were very similar to those found in Studies 1 and 2, with presentation durations that were very consistent.

#### Visual duration

Variability was intermediate between the results found in Studies 1 and 2.

#### SOA between auditory and visual onset

**Fig. 3:**
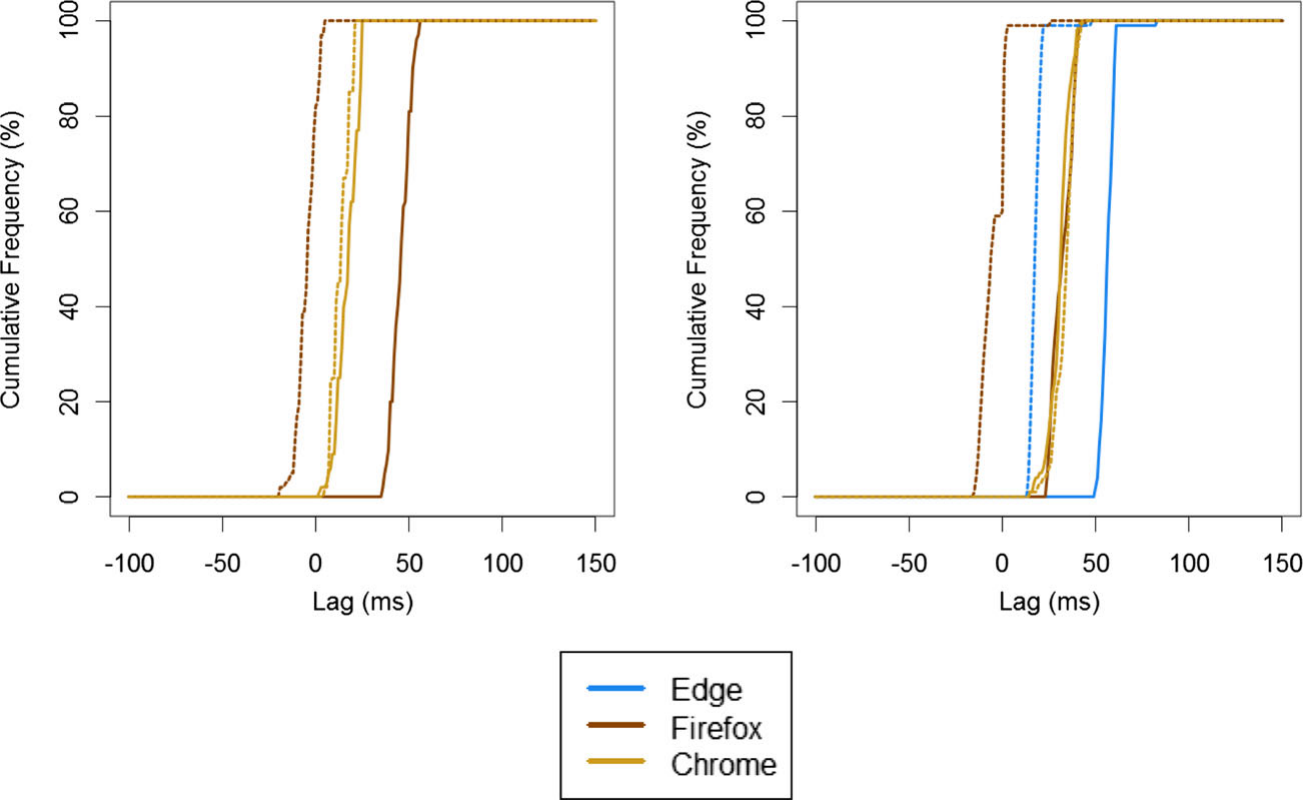
Cumulative frequency distribution of SOAs in Study 3 using the Web Audio API. Here, the left panel shows performance on the desktop (with no test for Microsoft Edge), and the right panel shows performance on the laptop. Solid lines indicate performance using mp3, and the dotted lines indicate performance using wav

The distribution of SOAs is given in Fig. 3. It appears that the use of wav files led to shorter latencies than did mp3 files, and so reduced the SOA. However, within system–browser variability and between system–browser variability were similar across the two formats. Similarly, when we compared the performance here using Web Audio with that using the very basic coding in Study 1, although SOAs were overall smaller using Web Audio, and the variability within system–browser combinations in the laptop condition was lower, variability across system–browser combinations was not reduced.

### Discussion

We attempted to reduce overall SOAs and, more importantly, the variability in SOAs across system–browser combinations by (a) using the wav audio format rather than mp3 and (b) using the Web Audio API. We found that using wav format reduced the overall SOAs but, disappointingly, did not reduce the overall variability in SOAs across system–browser combinations. The use of the Web Audio API on average reduced the SOAs slightly and reduced the within system–browser combination *SD*s for the lower-powered laptop. This second finding is comparable to Slote and Strand (2016) finding that *SD*s increased less under high processor load when the Web Audio method was used.

## Study 4

In Study 4, we used Web Audio in a slightly different way to schedule presentation of the stimuli. The basic approach is shown in the pseudocode below. The system works by looking ahead at every animation frame to see whether a sine wave should begin playing in the near future. If playback is due, its exact time is scheduled using the highly accurate audio clock. This look-ahead-quite-often-and-schedule-exactly approach is detailed in www.html5rocks.com/en/tutorials/audio/scheduling/.

1. Quite often, check to see whether a sine wave is due to be played soon. This is achieved by using requestAnimationFrame() to call a custom function schedule(). The method requestAnimationFrame() is designed to call a custom function before the next repaint of the screen, which will be 60 times a second for a 60-Hz display. But it is not run at millisecond-reliable times.
2. If a sine wave is due within the next 200 ms:
  - Create the sine wave using createBufferSource() on an AudioContext() context object.
  - Once the sine wave buffer source is created, attach a callback to hide the square to be run when the sine wave ends. This is done using the onended() method.
  - Schedule the sine wave buffer source to play at an exact time using the audio clock. The sine wave is scheduled to play at an exact time on the audio-card hardware using the start() method on the buffer source.
  - Finally, schedule a callback to display the square at the same time as the start of the sine wave, using the main JavaScript clock and the setTimeout() method. [It would be better if an onstart() method existed for the buffer source, so that the square onset could be scheduled using the audio clock. Unfortunately, the onstart() function does not exist.]

### Results and discussion

The full results can be seen in Table 4.

**Table 4:** Deviations from intended stimulus durations and synchronization by browser and system in Study 4, for desktop PC (left) and laptop (right)

	JavaScript Web Audio Desktop		JavaScript Web Audio Laptop		
	Firefox	Chrome	Edge	Firefox	Chrome
Auditory Duration					
Mean	+19.6 (*SD*)	+19.6 (0.60)	+25.2 (0.64)	+25.7 (0.77)	+25.9 (0.87)
Range	+18, +20	+18, +23	+24, +27	+25, +28	+25, +28
Visual Duration					
Mean (*SD*)	+92.1 (8.6)	+24.0 (10.3)	+67.8 (8.1)	+95.1 (20.8)	+22.8 (6.9)
Range	+74, +102	+7,+35	+57, +79	+73, +252	+6, +56
SOA (Auditory – Visual)					
Mean (*SD*)	+32.7 (6.1)	+5.2 (4.9)	+47.9 (5.2)	+26.3 (6.2)	+27.4 (5.1)
Range	+11, +45	–3, +11	+37, +59	+3, +38	+10, +36

Positive values for SOA indicate that audio lagged behind visual

#### Auditory duration

The results are very similar to those found in Study 3, with presentation durations being very consistent.

#### Visual duration

The results are again very similar to those found in Study 3—presentation durations are rather more variable

#### SOA between auditory and visual onset

**Fig. 4:**
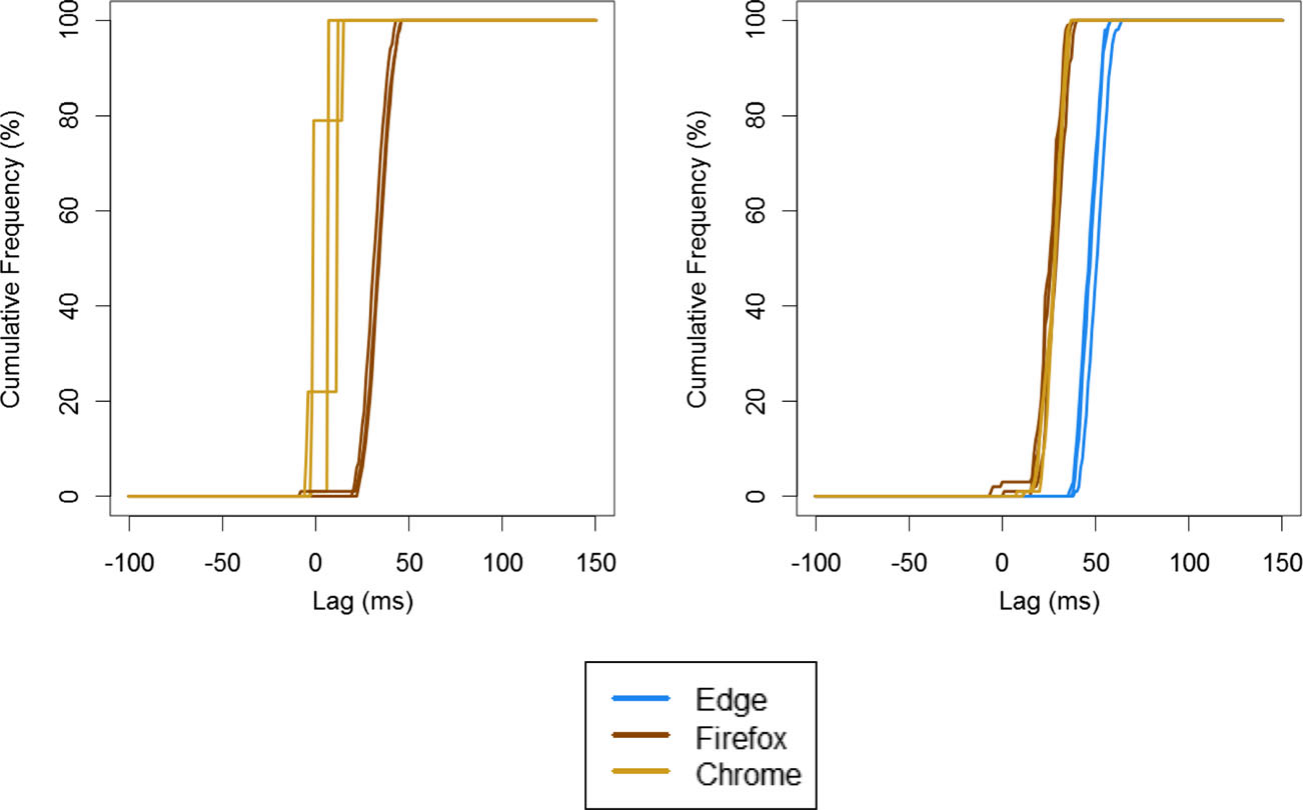
Cumulative frequency distribution of SOAs in Study 4 using the Web Audio API. Here, the left panel shows performance on the desktop (with no test for Microsoft Internet Explorer), and the right panel shows performance on the laptop. Three lines of a single color show test–retest consistency

The distribution of SOAs is given in Fig. 4. The results across repeats of the same system–browser combination are very consistent, but the discrepancy across browsers and systems is of comparable magnitude to that found in Study 3.

Overall, the results of Study 4 are similar to those in Study 3, with variability of around 40 ms in the mean SOAs of auditory and visual stimuli presented across different browsers and systems.

## General discussion

Across four studies, we examined the accuracy of auditory stimulus presentation and synchronization between auditory and visual stimuli over the Web. We used both JavaScript and Flash, two approaches to synchronizing stimuli, two audio formats, and two JavaScript methods for controlling presentation. We also tested three browsers on two computer systems. Overall, we were not able to synchronize auditory and visual presentation in a consistent way across different system–browser combinations. Across the six (3 Browsers × 2 Systems) setups we used, running the same code in a browser led to substantial variability of mean SOAs. Even in the conditions that were most consistent across system–browser combinations, we found that the SOAs varied by 40 ms. We would expect variability to be higher than this if a wider range of computers were to be used. As such, these findings should be treated as a minimum variability to expect across systems.

### Implications for cross-modal research

The results presented here pose problems for people wishing to run cross-modal experiments online. Running the same Flash or JavaScript code on different system–browser combinations leads to very different, often nonoverlapping, distributions of SOAs. If a study requires SOAs to be precise (say, <50 ms), we would caution against the use of the Web-based procedures we tested here. We also recommend that lab-based studies verify the SOA on the specific setup that they are using: Inaccuracies in cross-modal SOAs are not uniquely a result of testing online, and may occur to similar degrees in the lab (Babjack et al., 2015).

However, there may be ways of using Web-based methodologies for cross-modal research. For example, other ways of coding studies might allow Flash or JavaScript to control the timing of auditory onsets more accurately, or synching visual onset to auditory onset more effectively. Also, other ways of presenting auditory and visual stimuli might not have the same synchronization issues. Alternatively, the use of video, now reasonably well-supported in HTML5 and Flash, might allow for more accurate synchronization of auditory and visual stimuli. An alternative approach might be to allow participants to synchronize the auditory and visual onsets themselves—they might be allowed to adjust the onset of a visual stimulus so that it appears to them to coincide with the auditory stimulus. Of course, the researcher would not be able to know whether this was done accurately.

Finally, it might be possible to tailor the presentation onsets for a given system–browser combination. For example, a researcher might be able to write code that records the actual lag between auditory and visual onsets and adjusts the stimulus presentation to compensate for the lag for a given user on a given system–browser combination. One low-tech approach would be, at the start of an experiment, to ask participants to place their microphone by their speaker or headphones and have the system record the lag between executing the command to start the audio and detecting the auditory input via the microphone. Both Flash and JavaScript Web Audio allow microphone input, with users’ permission, and this approach would be particularly effective at minimizing errors when participants have to give verbal responses using a voice key.

### Implications for auditory response time research

We did not measure directly the accuracy of RT measurements for auditory stimuli. However, we can estimate it by combining the results here with those of earlier work. We previously (Reimers & Stewart, 2015) measured RT accuracy to visual stimuli using the Black Box Toolkit. We previously found that the measured visual RTs using Flash or JavaScript were 30–100 ms longer than the actual RTs. Some of the extra duration was due to the lag between pressing a key and the keypress being detected by JavaScript or Flash. However, some of the extra duration was due to the fact that the timer starts when the command to present the stimulus is executed, which is several milliseconds later. Since we now know that auditory stimulus onsets have an even longer lag than visual stimulus onsets, we can predict the degree of overestimation of auditory RTs. From the research presented here, if stimuli were simply set to play, and a timer to measure RTs were started concurrently, then the overestimation of RTs would be 70–200 ms. This can be reduced by an appropriate choice of implementation, such as using JavaScript rather than Flash, and within JavaScript by using Web Audio scheduling. Whichever method is used, the variability in within-system auditory onset lag will still be relatively low (*SD*s of a maximum of 15.1 ms, which includes the variability in visual presentation onset as well as that for auditory onset). As we and others have shown (Brand & Bradley, 2012; Damian, 2010; Reimers & Stewart, 2015; Ulrich & Giray 1989), the effects of small amounts of extra noise such as those seen here will have minimal effects on the results obtained from typical multitrial experiments. Furthermore, it appears that variability and overestimation may be reduced slightly by using the Web Audio method (as Slote & Strand, 2016, demonstrated), if experimenters are willing to exclude participants who use Internet Explorer. As such, the use of auditory stimuli in Web-based RT research seems feasible.

## Electronic supplementary material

Below is the link to the electronic supplementary material.

ESM 1 (ZIP 1310 kb)

## Footnotes

1 We chose to use a sine wave as our auditory stimulus for simplicity, and consistency with previous research. With hindsight, we think we should have used white noise or similar. This would have prevented an audible “pop” at the end of the stimulus presentation, presumably because the stimulus finished at an arbitrary—nonzero—point in its sinusoidal cycle, and the speaker would then have returned to its central zero position. This may explain why measured stimulus duration was greater than 1,000 ms. However, the use of a sine wave should not affect measured stimulus onsets or variability in lags between auditory and visual onsets.
