# Supplementary material for: Auditory presentation and synchronization in Adobe Flash and HTML5/JavaScript Web experiments
Source: Behav Res Methods. 2016 Jul 15;48(3):897–908. doi: 10.3758/s13428-016-0758-5 (PMC5003904; doi:10.3758/s13428-016-0758-5)
Supplement: Supplementary file 1 — (ZIP 1310 kb) [file 13428_2016_758_MOESM1_ESM.zip › Code/JavaScript_code/jswa2-js.html]

# Experiment

Audio playback accuracy.

ISI duration in seconds:

Start

Your browser does not support the audio element.

Play 1000 Hz 1000 ms sine wave Stop

This is the Web Audio API version, using the Web Audio Clock and requestAnimationFrame().

See http://www.html5rocks.com/en/tutorials/audio/scheduling/ and http://catarak.github.io/blog/2014/12/02/web-audio-timing-tutorial/.

Works by schedule() setting itself up to be called repeatedly by requestAnimationFrame().

Basically, schedule() is called every retrace. If the time to play the tone is less than planAhead seconds away, schedule the tone to start at a specific time on the audio clock, and set up a call cack to hide the square when the tone finishes. Because there is no callback for when a tone starts, also schedule a square to appear at the same time using standard setTimeout(). Then set up a new time for the next tone.

# Thank you!

The test is over.
