# Supplementary material for: Auditory presentation and synchronization in Adobe Flash and HTML5/JavaScript Web experiments
Source: Behav Res Methods. 2016 Jul 15;48(3):897–908. doi: 10.3758/s13428-016-0758-5 (PMC5003904; doi:10.3758/s13428-016-0758-5)
Supplement: Supplementary file 1 — (ZIP 1310 kb) [file 13428_2016_758_MOESM1_ESM.zip › Code/JavaScript_code/jswa-js.html]

# Experiment

Audio playback accuracy.

ISI duration in ms:

Start

Your browser does not support the audio element.

Play 1000 Hz 1000 ms sine wave Stop

This is the Web Audio API version.

Works by playing a 1000 ms tone. The onset of the square is the line of code immediately after the .start() call to start playing. The offset of the square and the start of the ISI are bound to the "ended" event. The function bound to ended also make a new tone (can't reuse with Web Audio) and starts the ISI using a callback timer. The callback timer then plays the tone and displays the square.

# Thank you!

The test is over.
