# Supplementary material for: Auditory presentation and synchronization in Adobe Flash and HTML5/JavaScript Web experiments
Source: Behav Res Methods. 2016 Jul 15;48(3):897–908. doi: 10.3758/s13428-016-0758-5 (PMC5003904; doi:10.3758/s13428-016-0758-5)
Supplement: Supplementary file 1 — (ZIP 1310 kb) [file 13428_2016_758_MOESM1_ESM.zip › Code/JavaScript_code/sep-js.html]

# Experiment

Audio playback accuracy.

ISI duration in ms:

Stimulus duration in ms:

Start

Your browser does not support the audio element.

Play 1000 Hz 1000 ms sine wave Stop

Works by playing a 1000 ms tone. The code to display the square follows immediately after the code to display the sound. While the sound just runs out and finishes, the square offset is triggered by a timer. Finally the trial starts again after another timer for the ISI expires.

# Thank you!

The test is over.
