# Supplementary material for: Auditory presentation and synchronization in Adobe Flash and HTML5/JavaScript Web experiments
Source: Behav Res Methods. 2016 Jul 15;48(3):897–908. doi: 10.3758/s13428-016-0758-5 (PMC5003904; doi:10.3758/s13428-016-0758-5)
Supplement: Supplementary file 1 — (ZIP 1310 kb) [file 13428_2016_758_MOESM1_ESM.zip › Code/JavaScript_code/sync-js.html]

# Experiment

Audio playback accuracy.

ISI duration in ms:

Start

Your browser does not support the audio element.

Play 1000 Hz 1000 ms sine wave Stop

Works by playing a 1000 ms tone. The onset of the square is bound to the detection of the "play" event (which seems to work better than putting the display square code at the same place as code to start playing the sound). The offset of the square and the start of the ISI are bound to the "ended" event. The start of the tone is bound to the callback for the end of the ISI duration.

This is the "playing" version, where the square display is bound to the "playing" event.

# Thank you!

The test is over.
