# Supplementary figures and images for: Auditory presentation and synchronization in Adobe Flash and HTML5/JavaScript Web experiments
Source: Behav Res Methods. 2016 Jul 15;48(3):897–908. doi: 10.3758/s13428-016-0758-5 (PMC5003904; doi:10.3758/s13428-016-0758-5)

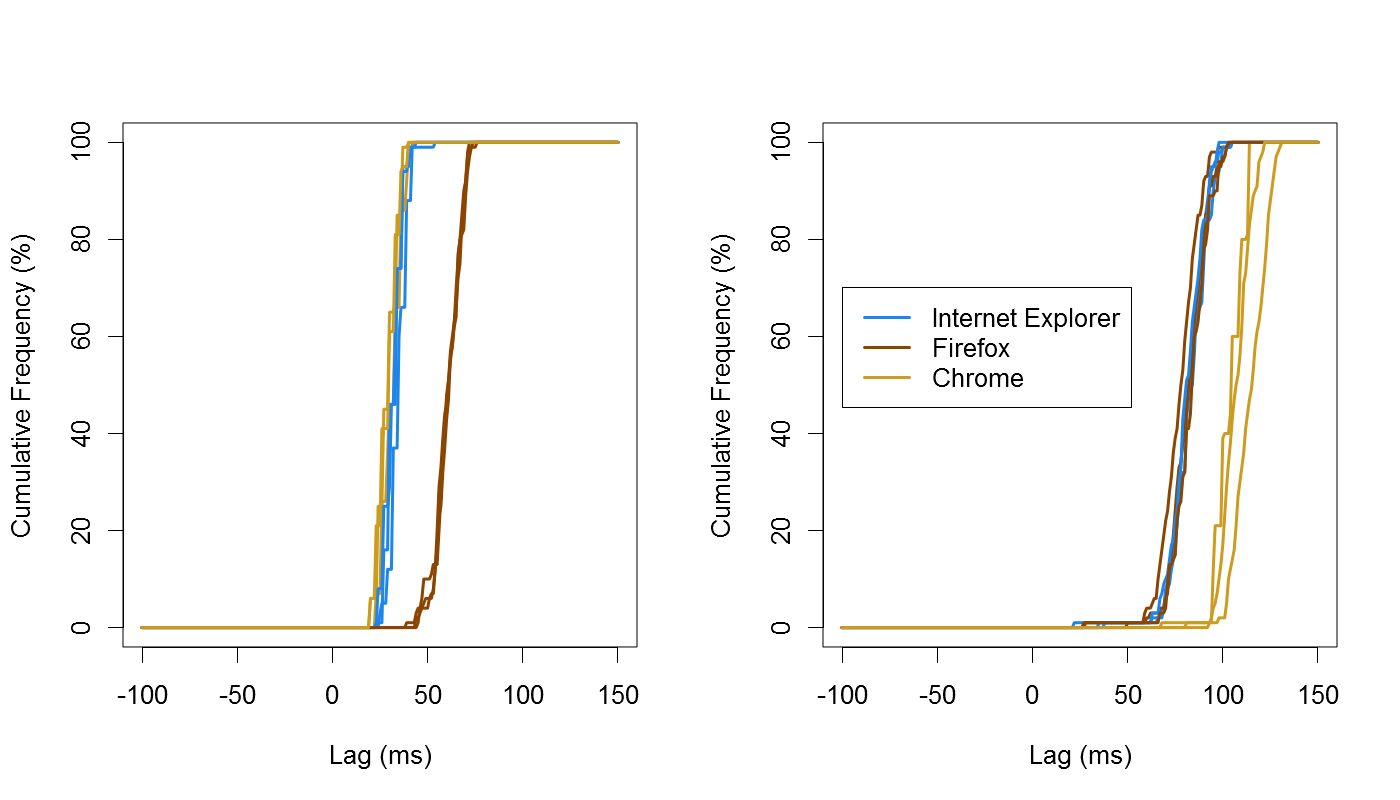

Supplement: Supplementary file 1 — (ZIP 1310 kb) [file 13428_2016_758_MOESM1_ESM.zip › Data/S1/Fig1.png]

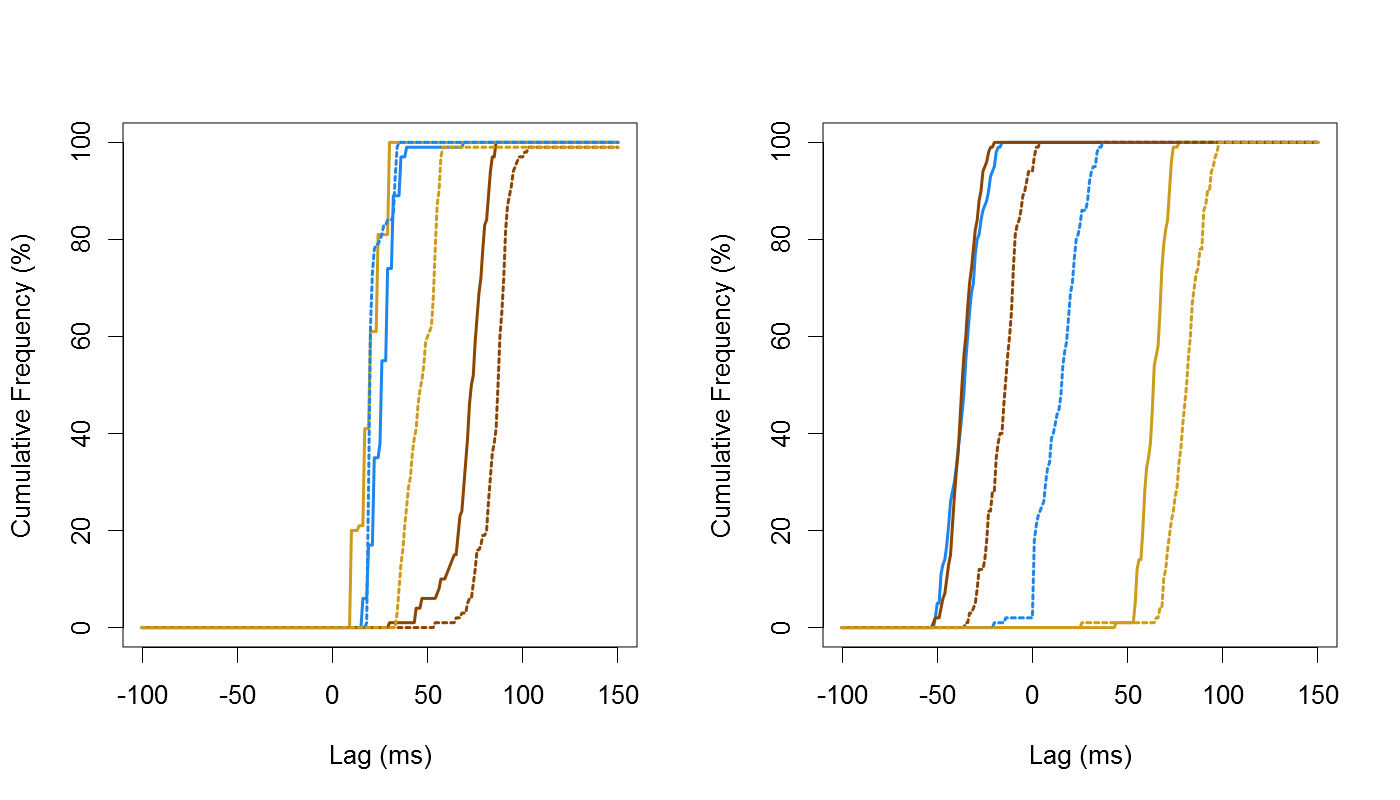

Supplement: Supplementary file 1 — (ZIP 1310 kb) [file 13428_2016_758_MOESM1_ESM.zip › Data/S2/Fig2.png]

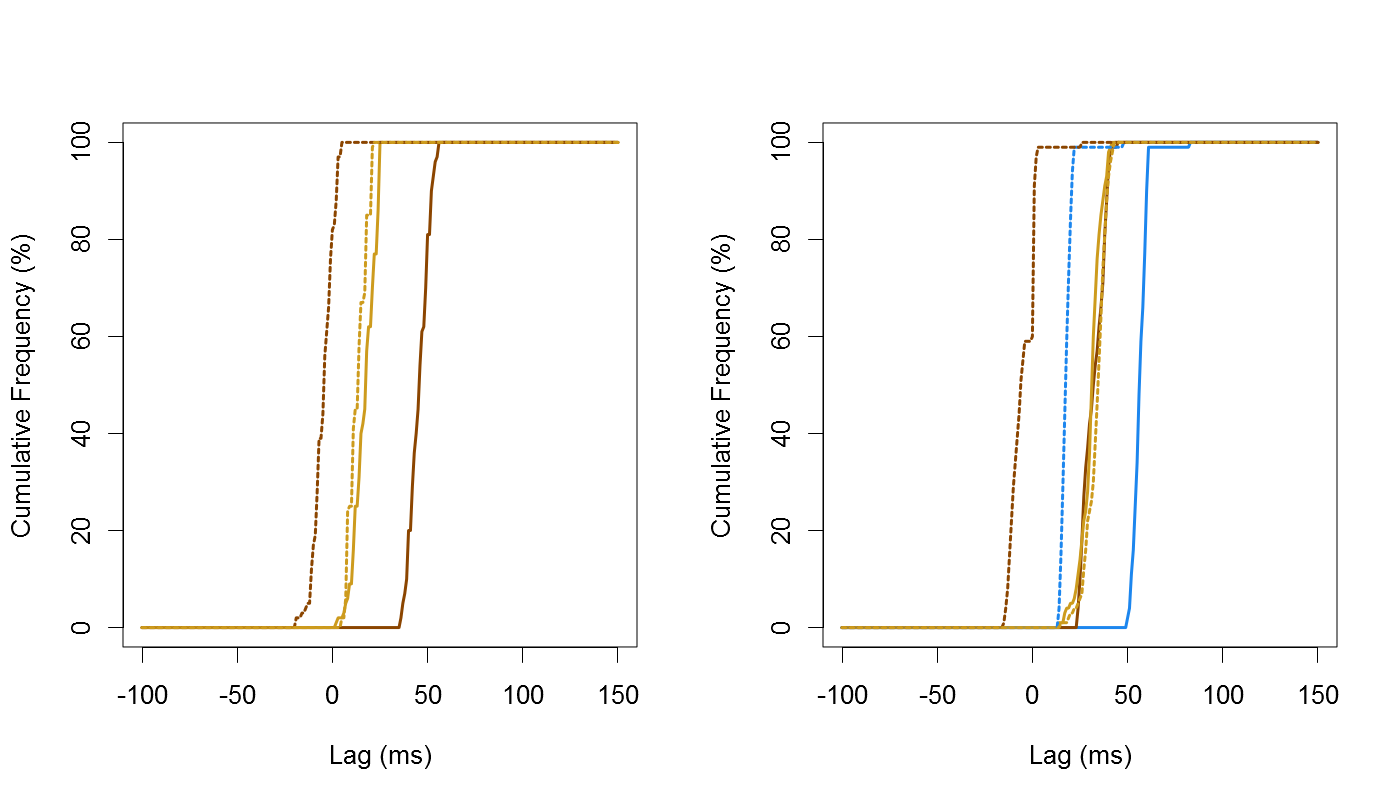

Supplement: Supplementary file 1 — (ZIP 1310 kb) [file 13428_2016_758_MOESM1_ESM.zip › Data/S3/Fig3.png]

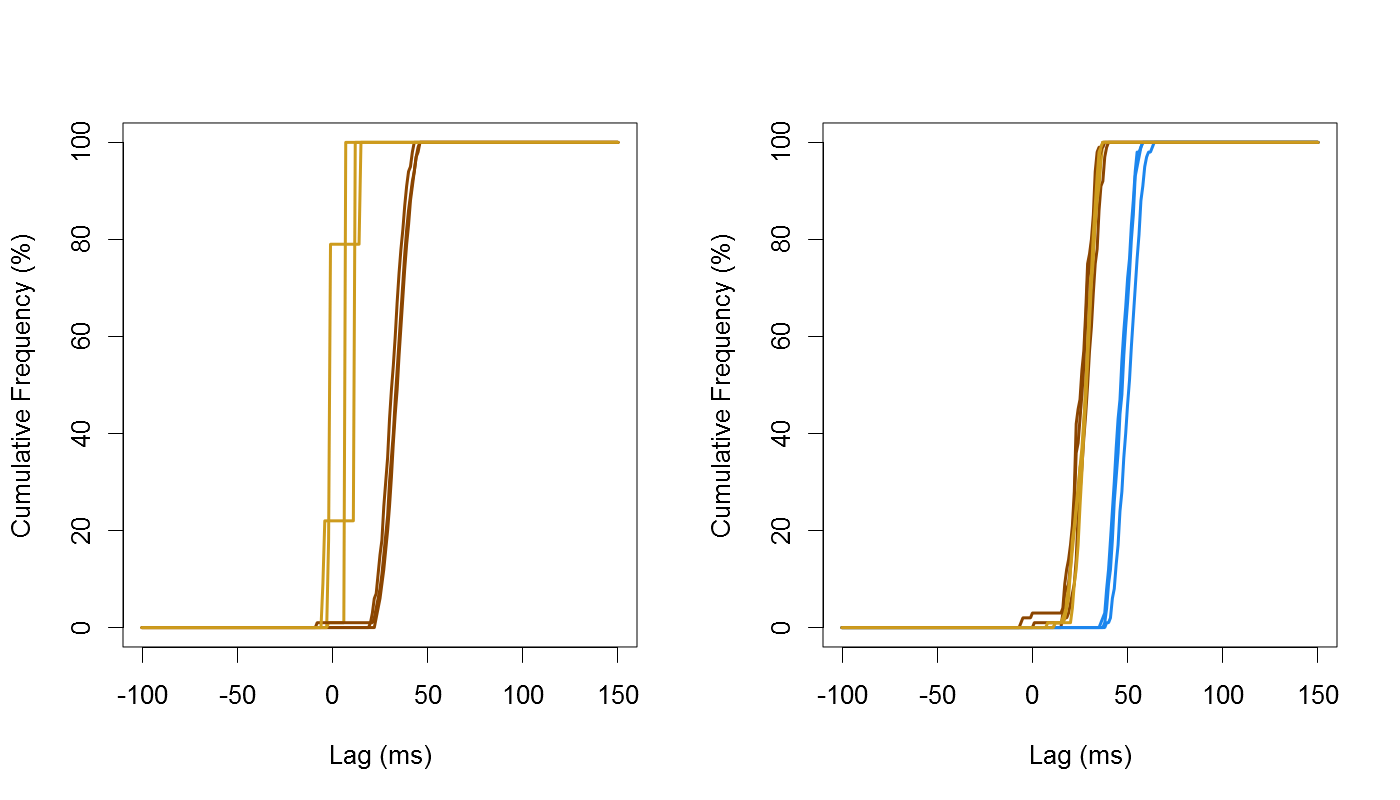

Supplement: Supplementary file 1 — (ZIP 1310 kb) [file 13428_2016_758_MOESM1_ESM.zip › Data/S4/Fig4.png]
